# Predominant Antibody Deficiency and Risk of Microscopic Colitis: a Nationwide Case–Control Study in Sweden

**DOI:** 10.1007/s10875-023-01499-3

**Published:** 2023-05-10

**Authors:** Daniel V. DiGiacomo, Bjorn Roelstraete, Lennart Hammarström, Jocelyn R. Farmer, Hamed Khalili, Jonas F. Ludvigsson

**Affiliations:** 1 Clinical and Translational Epidemiology Unit, Mongan Institute, Massachusetts General Hospital, Boston, MA USA; 2 Division of Rheumatology, Allergy and Immunology, Massachusetts General Hospital, Harvard Medical School, Boston, MA USA; 3 Sachs’ Children and Youth Hospital, Stockholm South General Hospital, Stockholm, Sweden; 4 Division of Clinical Immunology and Transfusion Medicine, Department of Laboratory Medicine, Karolinska Institutet, Stockholm, Sweden; 5 Division of Allergy and Inflammation, Beth Israel Lahey Health, Harvard Medical School, Boston, MA USA; 6 Division of Gastroenterology, Massachusetts General Hospital, Harvard Medical School, Boston, MA USA; 7 Division of Clinical Epidemiology, Department of Medicine, Karolinska Institutet, Stockholm, Sweden; 8 Department of Medical Epidemiology and Biostatistics, Karolinska Institutet, Stockholm, Sweden; 9 Department of Pediatrics, Orebro University Hospital, Orebro, Sweden; 10 Department of Medicine, Columbia University College of Physicians and Surgeons, New York, NY USA

**Keywords:** Predominant antibody deficiency, Common variable immune deficiency, Microscopic colitis, Nationwide case–control study

## Abstract

**Purpose:**

Predominant antibody deficiency (PAD) disorders, including common variable immunodeficiency (CVID), have been linked to increased risk of gastrointestinal infections and inflammatory bowel diseases. However, there are limited data on the relationship between PAD, specifically CVID, and risk of microscopic colitis (MC).

**Methods:**

We performed a nationwide case–control study of Swedish adults with MC diagnosed between 1997 and 2017 (*n* = 13,651). Data on biopsy-verified MC were retrieved from all of Sweden’s pathology departments through the Epidemiology Strengthened by histoPathology Reports in Sweden (ESPRESSO) study. We defined predominant antibody deficiency using International Union of Immunologic Societies (IUIS) phenotypic classification. Individuals with MC were matched to population controls by age, sex, calendar year, and county. We used logistic regression to estimate adjusted odds ratios (aORs) and 95% confidence intervals (CIs).

**Results:**

The prevalence of PAD in MC was 0.4% as compared to 0.05% in controls. After adjustment for potential confounders, this corresponded to an aOR of 7.29 (95%CI 4.64–11.63). The magnitude of the association was higher for CVID (aOR 21.01, 95% 5.48–137.44) compared to other antibody deficiencies (aOR 6.16, 95% CI 3.79–10.14). In exploratory analyses, the association between PAD and MC was particularly strong among males (aOR 31.73, 95% CI 10.82–135.04).

**Conclusion:**

In this population-based study, predominant antibody deficiency was associated with increased risk of MC, particularly among males. Clinicians who encounter these patients should consider a detailed infectious history and screening for antibody deficiency.

**Supplementary Information:**

The online version contains supplementary material available at 10.1007/s10875-023-01499-3.

## Introduction

Microscopic colitis (MC) is an inflammatory bowel disease (IBD) of the large intestine that includes two histologic subtypes, collagenous colitis (CC), and lymphocytic colitis (LC). Diagnosis of MC requires histologic confirmation of an inflammatory infiltrate in the colon. Criteria are partitioned by MC subtype, with LC defined by the presence of > 20 lymphocytes/100 epithelial cells and CC by an expansion of subepithelial collagen fiber (> 10 µm in diameter) with lymphocytic infiltration [1]. MC predominantly affects older individuals accounting for up to 20% of chronic watery diarrhea [2–4]. Its pathogenesis is largely unknown but is associated with HLA ancestral haplotype 8.1, pathologic T cell activation, and intestinal dysbiosis [5–9]. Risk of MC is increased in those with prior gastrointestinal (GI) infection, autoimmunity, or who take certain medications [10–13]. Individuals with MC have increased infection-related mortality, further demonstrating the impact of a dysregulated immune response in this disease [14].

Predominant antibody deficiencies (PAD) are defined by low levels or low function of circulating antibodies. Within this classification, common variable immunodeficiency (CVID) is the most common symptomatic primary immunodeficiency worldwide. The prevalence of CVID is up to 1:25,000, and it most frequently affects those aged 20–40 years, with an equal sex predisposition, although earlier age of onset and more severe disease course have been reported in males as compared to females [15–19]. CVID is a disorder of increased infectious risk that results from impaired and dysregulated immunity. Diagnosis requires low IgG combined with low IgA/IgM, impaired vaccine response, and the exclusion of secondary causes. While individuals with CVID typically present with recurrent or atypical infections, more than 30% demonstrate non-infectious, autoinflammatory GI manifestations [20–22].

Chronic diarrhea is the most common GI complication in CVID, stemming from either infection or autoinflammatory involvement in the small bowel or colon. In particular, non-infectious GI complications are associated with decreased quality of life and a threefold increased risk of death [23, 24]. Despite this, most prior studies of GI complications in CVID have largely reflected single or multi-center experiences and there have been no population-based studies to systematically assess risk of various inflammatory conditions of the GI tract in patients with PAD. Several lines of evidence suggest that MC may be a common cause of inflammatory diarrhea in patients with CVID. First, the predominant GI histopathologic findings in CVID include aggregates of CD8 + T cell lymphocytes and increased Th1 cytokines in the epithelium and lamina propria, which are considered hallmark histologic findings in MC [25, 26]. Second, recurrent GI and pulmonary infections which are risk factors for MC are among the most common clinical manifestations of CVID [13, 27]. Lastly, at least one recent study from Finland has shown that among 163 CVID patients and GI manifestations, up to 10% developed MC [28]. We, therefore, set out to examine the relationship between PAD, particularly CVID, and risk of MC using a nationwide histopathology cohort in Sweden.

## Methods

### Study Population

#### Ascertainment of MC Cases

The methodology for identifying MC cases in Sweden has been previously described by our group [10, 29, 30]. Briefly, the ESPRESSO (Epidemiology Strengthened by histopathology Reports in Sweden) cohort is a nationwide histopathology study. It incorporates data from GI biopsies from all 28 pathology departments in Sweden from January 1965-April 2017 [31]. The information provided in each pathology report from ESPRESSO includes personal identity number, biopsy date, anatomic location, and morphology according to Systematized Nomenclature of Medicine (SNOMED) coding system (CC: M40600, LC: M47170). This study examined all MC cases from 1997 to 2017, as routine diagnosis of MC was not present until the late 1990s. The method of confirming MC diagnosis has been previously validated, with a positive predictive value of 95% when comparing SNOMED codes to medical charts [32]. Time of MC diagnosis was defined as the first available biopsy with indicative histopathologic findings. Given the complicated nature of histopathologic findings in patients with CVID, we also individually reviewed available free-text pathology reports of those with both CVID and MC. This study was performed with approval from the Stockholm Ethics Review Board. Informed consent was waived given the register-based approach [33].

#### General Population Controls

Individuals with MC were matched to 5 population comparators without MC or any prior GI biopsy derived from the Swedish Total Population Register. Matching was based on age, sex, calendar year, and county at diagnosis and performed through the use of a personal identity number allotted to all Swedish residents [34].

#### Sibling Controls

Full siblings to the MC patients were identified utilizing the Swedish Multigeneration Register, which is a component of the Total Population Register [34].

### Ascertainment of Immunodeficiency

The Swedish Patient Register includes nationwide individual-level data on inpatient and specialized outpatient encounters. Patients with PAD were identified using International Classification of Diseases (ICD) 10^th^ Revision coding for CVID or immunodeficiency with predominant antibody defects and defined according to the 2022 updated International Union of Immunologic Societies (IUIS) classification [35]. Patients are herein referred to as having PAD, which is further subdivided into those with hereditary hypogammaglobulinemia, which consisted of a single patient in this analysis, and those greater than four years of age with CVID (‘CVID’), or those with other predominant antibody deficiencies (“Other PAD”) (Table S1). In order to maintain consistent terminology we employed exact ICD 10^th^ Revision phrasing. This includes the coding of hereditary hypogammaglobulinemia, which refers to autosomal recessive and X-linked cases of agammaglobulinemia. This also includes non-familial hypogammaglobulinemia, which is a non-specific and heterogeneous term. For participants with multiple different ICD 10^th^ Revision PAD codes, the specific diagnosis closest to the date MC diagnosis was chosen. Diagnostic coding in the Swedish Patient Register has been validated with positive predictive values generally ranging between 85 and 95% [36].

### Ascertainment of Other Covariates

The Total Population Register contains demographic information on age, sex, country of birth, and county of residence [33]. The Swedish Prescribed Drug Register was utilized to gather data on immunoglobulin therapy (Table S2) [37]. This Register contains information on all dispensed medications starting on July 1, 2005, based on the Anatomical Therapeutic Chemical (ATC) classification system. Participants were defined as receiving replacement immunoglobulin therapy if they had a record of administration prior to MC diagnosis or control index date. Information on the comorbidities relevant to CVID and MC was collected from the Total Swedish Patient Register. This included a history of GI infections, and/or sinopulmonary infections (Table S3). Additional immune-related diagnoses were also collected (Table S4), as were causes of secondary immunodeficiency (Table S5). Those with 1 or more recorded encounters were considered to have a positive co-morbidity history.

### Statistical Analysis

**Fig. 1:**
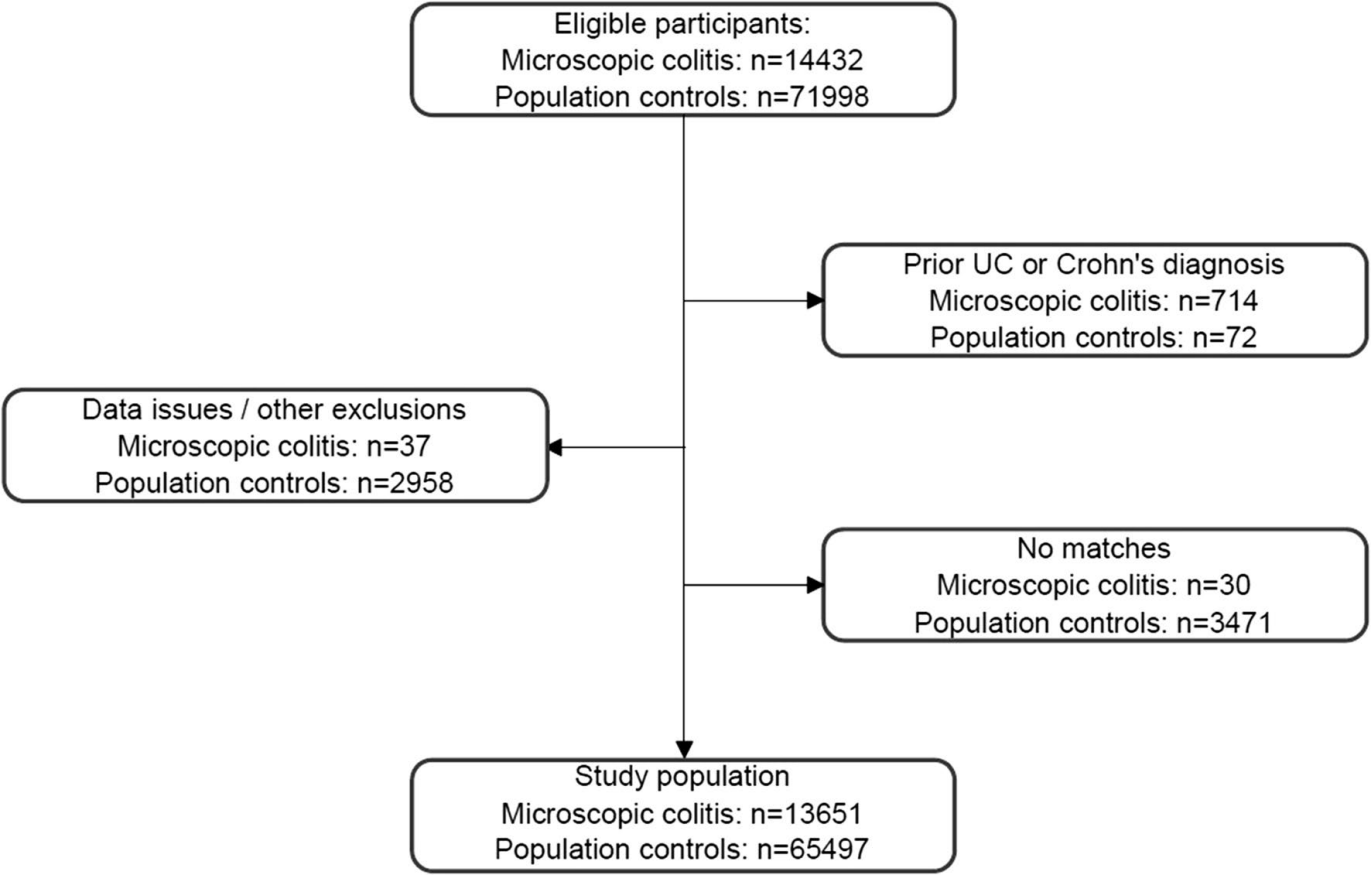
Flowchart of eligible study participants. UC ulcerative colitis

We conducted a case–control study of all individuals in Sweden who were diagnosed with MC from January 1, 1997, until December 1, 2017, matched to 5 population controls by age, sex, calendar year, and county of residence. Individuals with and without MC were modeled for previous diagnoses of CVID or other PAD. Those with inflammatory bowel disease (Crohn’s disease and ulcerative colitis), as previously defined in Swedish population data, were excluded from this study at the time of the index date (Fig. 1) [38]. For our primary analysis, we used logistic regression modeling to estimate the odds ratio (OR) and 95% confidence interval (CI) and adjusted our models for matching factors including age, sex, county, and calendar year as well as other potential confounders including immune-mediated diseases.

We conducted several sensitivity and exploratory analyses to further characterize the relationship between CVID, other predominant antibody deficiencies, and risk of MC. First, we sought to determine if our findings were mediated by GI and/or sinopulmonary infections in the five years prior to MC by further adjusting our models for these factors. Next, we investigated whether the relationship was affected by well-captured relevant non-infectious immune-related co-morbidities diagnosed prior to MC. We also redefined our primary exposure as those with either IgA deficiency only, nonfamilial hypogammaglobulinemia only, or CVID excluding hereditary hypogammaglobulinemia to examine our observed associations according to specific types of immunodeficiency. To account for potential sources of misclassification in PAD, we then considered the impact of exposures associated with secondary immunodeficiency. Secondary immunodeficiency may stem from decreased immunoglobulin production (immunosuppression, malignancy, malnutrition, human immunodeficiency virus), or increased immunoglobulin losses (via GI tract, renal or skin) [39]; therefore, we excluded individuals with human immunodeficiency virus, myeloid or lymphoid malignant and pre-malignant disorders, and intestinal malabsorption (a surrogate for protein-losing enteropathy), nephrotic syndrome, severe burns, or malnutrition (Table S3, Table S5). Finally, we explored the relationships across the different strata of sex, age at diagnosis of CVID (< 50 vs ≥ 50 years), time from PAD diagnosis to diagnosis of MC (< 1 year, 1 to < 5 years, ≥ 5 years) and different calendar periods (1997–2006, 2007–2011, 2012–2017). Lastly, to account for shared genetic and early environmental factors, we used unaffected sibling comparators to examine the association between PAD and risk of MC (Table S6).

Statistical analyses were performed utilizing R version 3.5.1 (R Foundation for Statistical Computing, Vienna, Austria). A *P* value of < 0.05 was deemed to be statistically significant.

## Results

### Participant Characteristics

After exclusion, we included 13,651 individuals with MC (4410 CC, 9241 LC) matched to 65,497 population controls. The mean age of diagnosis of MC was 60.6 years (standard deviation 16.7), with the majority of cases being older than 50 years of age (76.6%) and female (72.3%). Most participants with MC were diagnosed after 2007 (67.4%) (Table 1).

**Table 1:** Baseline characteristics of cases of microscopic colitis and general population controls

	Microscopic colitis	Population controls
	*n* [%]	*n* [%]
Total	13,651 [100.0]	65,497 [100.0]
Sex, female	9872 [72.3]	47,463 [72.5]
Age at start of follow-up, years		
Mean [SD]	60.6 [16.7]	60.0 [16.6]
Median [IQR]	63.0 [51.0–73.0]	63.0 [50.0–72.0]
< 50 years	3194 [23.4]	15,791 [24.1]
≥ 50 years	10,457 [76.6]	49,706 [75.9]
Time from PAD to MC/index date, years		
Mean [SD]	6.7 [5.8]	7.1 [4.9]
Median [IQR]	5.0 [2.1–9.7]	5.4 [3.3–9.6]
< 1 years	8 [16.0]	2 [6.7]
1 < 5 years	17 [34.0]	12 [40.0]
≥ 5 years	25 [50.0]	16 [53.3]
Year of start of follow-up		
1997–2001	1329 [9.7]	6458 [9.9]
2002–2006	3111 [22.8]	14,994 [22.9]
2007–2011	4592 [33.6]	22,007 [33.6]
2012–2017	4619 [33.8]	22,038 [33.6]

*IQR* interquartile range, *MC* microscopic colitis, *PAD* predominant antibody deficiency, *SD* standard deviation

### PAD and CVID Specifically Are Associated with Increased Risk of MC

We investigated the relationship between PAD and risk of MC. Of those with MC, 50 individuals had a prior diagnosis of PAD amounting to a population frequency of 1 in 273 (0.37%), which was significantly higher than population controls (0.046%, 1 in 2183 individuals). After adjusting for matching factors of age, sex, calendar year, county and immune-mediated disease this translated to an adjusted OR [aOR] of 7.29 (95% CI 4.64–11.63), with the association being stronger among those with CVID (aOR 21.00; 95% CI 5.47–137.42), compared to other PAD (aOR 6.16; 95% CI 3.79–10.14) (Table 2). The association between PAD and MC remained significant across histologic subtypes of MC, CC (aOR 7.16 (95%CI 3.10–17.35)) and LC (aOR 7.46 (95%CI 4.37–13.03) (Table S7).

**Table 2:** Association between predominant antibody deficiency and risk of microscopic colitis

	MC exposed (*N*)	Controls exposed (*N*)	aOR [95% CI]
PAD	50/13,651	30/65,497	7.29 [4.64–11.63]
CVID	10/13,650	2/65,497	21.00 [5.47–137.42]
Other PAD	39/13,650	28/65,497	6.16 [3.79–10.14]

aOR adjusted for age, sex, year, county, and immune-mediated disease

*CI* confidence interval, *CVID* common variable immunodeficiency, *MC* microscopic colitis, *OR* odds ratio, *PAD* predominant antibody deficiency

### Sensitivity Analyses

We considered several sensitivity analyses (Table 3). First, we evaluated the possibility that secondary immunodeficiency may lead to exposure misclassification. After excluding those with secondary immunodeficiency prior to the index date the association between PAD and risk of MC remained significant (aOR 6.9; 95%CI 4.31–11.24). Second, we defined cases of PAD as those who had received replacement immunoglobulin therapy and therefore limited our analysis to after July 1, 2006, which is 1 year after the prescribed drug register became available in Sweden. Similar to our main analysis, PAD with immunoglobulin therapy was associated with an increased risk of MC (aOR 3.11; 95%CI 2.02–4.73), albeit the magnitude of the association was smaller. Third, we excluded participants with hereditary hypogammaglobulinemia from our definition of PAD as these cases are enriched for monogenetic defects which may have accounted for the observed association. After excluding those with this diagnosis, the relationship between PAD and MC remained statistically significant (aOR 7.12; 95%CI 4.53–11.38). Fourth, we examined the associations with non-familial hypogammaglobulinemia, given its diagnostic heterogeneity (aOR 6.49; 95%CI 2.82–15.72), and IgA deficiency (aOR 17.64; 95%CI 7.68–47.72), given its involvement in mucosal immunity and observed similar results. Lastly, we considered the possibility that cases of MC among PAD patients were miscoded by pathologists and therefore reviewed their histology reports. Of 50 MC cases with prior diagnosis of PAD, texts from histology report were available for 21 cases (42%). Among these cases, 20/21 (95%) had clear features of MC based on the pathologists’ comments and the remaining one report had non-specific colitis features suggesting CVID-related indeterminate colitis.

**Table 3:** Association between predominant antibody deficiency and microscopic colitis across several sensitivity analyses

	MC exposed (N)	Controls exposed (N)	aOR [95% CI]
Excluding secondary immunodeficiency	45/13,463	28/65,092	6.9 [4.31–11.24]
Redefine PAD diagnosis			
PAD + replacement immunoglobulin	36/9555	55/45,689	3.11 [2.02–4.73]
PAD excluding hereditary hypogammaglobulinemia	49/13,651	30/65,497	7.12 [4.53–11.38]
Non-familial hypogammaglobulinemia	14/13,651	9/65,497	6.49 [2.82–15.72]
IgA deficiency	25/13,651	6/65,497	17.64 [7.68–47.72]

aOR adjusted for age, sex, year, county, and immune-mediated disease

*CI* confidence interval, *MC* microscopic colitis, *NA* not applicable, *OR* odds ratio, *PAD* predominant antibody deficiency

### Exploratory Analyses

In exploratory analyses, we examined the relationship between PAD and MC across several different strata defined by sex, age of diagnosis, years from PAD to MC diagnosis, and calendar period. The prevalence of PAD in males with MC was 1 in 189 individuals (0.53%), compared to females, 1 in 329 individuals (0.30%). Specifically, we observed evidence for effect modification by sex (P_interaction_ = 0.0047) with aOR of MC for males with PAD 31.72 (95%CI 10.82–135.03), as compared to aOR 4.68 (95%CI 2.76–7.97) in females with PAD (Table 4). Those < 50 years had a higher risk of MC (aOR 12.68; 95%CI 5.19–35.50) compared to those ≥ 50 years of age (aOR 5.93; 95%CI 3.50–10.19) although the formal statistical testing for the presence of interaction was not significant (P_interaction_ = 0.073). We then examined the relationship between time of PAD diagnosis to index date. Although, the magnitude of effect estimate for risk of MC was highest in those diagnosed with PAD within 1 year (aOR 19.82; 95%CI 4.95–131.61), the risk remained elevated for 1 < 5 years (aOR 6.30; 95%CI 3.01–13.59) and ≥ 5 years (aOR 6.50; 95%CI 3.48–12.50) before index date (P_interaction_ = 0.40). There were no significant differences in PAD and risk of MC according to different calendar period (Table 4). When stratifying between those with CVID and other PAD, these relationships generally remained the same (Table S8, Table S9). We also explored the possibility that the association between PAD and risk of MC may be mediated through recurrent infections and therefore further adjusted our main analyses for the most common infections in PAD including GI and sinopulmonary infections. The aOR of MC did not significantly change when accounting for GI (aOR 7.49; 95%CI 4.76–11.97) or sinopulmonary (aOR 7.13; 95%CI 4.54–11.38) infections. Lastly, we considered the possibility that our observed associations may me related to shared genetics or early environmental factors and therefore examined our associations using unaffected siblings as controls. The aOR of developing MC were 3.6 [95% CI 1.97–6.91] with PAD, and 5.00 [95%CI 1.21–20.65] with CVID specifically, when compared to unaffected siblings.

**Table 4:** Association between predominant antibody deficiency and microscopic colitis across selected strata

	MC exposed (N)	Controls exposed (N)	aOR [95% CI]
Total	50	30	7.29 [4.64–11.63]
Sex			
Males	20/3779	3/18,034	31.72 [10.82–135.03]
Females	30/9872	27/47,463	4.68 [2.76–7.97]
Age at MC diagnosis/matching, years			
≤ 50 years	17/3383	6/16,756	12.68 [5.19–35.50]
> 50 years	33/10,268	24/48,741	5.93 [3.50–10.19]
Years exposed before MC diagnosis/matching			
< 1 years	8/13,609	2/65,469	19.82 [4.95–131.61]
1 < 5 years	17/13,618	12/65,479	6.30 [3.01–13.59]
≥ 5 years	25/13,626	16/65,483	6.50 [3.48–12.50]
Year of MC diagnosis/matching			
1997–2001	0/1329	1/6458	NA
2002–2006	11/3111	10/14,994	4.68 [1.95–11.36]
2007–2011	11/4592	8/22,007	6.39 [2.57–16.62]
2012–2017	28/4619	11/22,038	11.03 [5.61–23.28]

aOR adjusted for age, sex, year, county, and immune-mediated disease

*CI* confidence interval, *MC* microscopic colitis, *NA* not applicable, *OR* odds ratio

## Discussion

Through a nationwide case–control study, we found a significant increase in risk of MC in those with PAD. The association between PAD and MC was stronger with CVID subtype and among males. Interestingly, recurrent GI or sinopulmonary infections did not mediate these associations. Our findings were robust across several sensitivity analyses.

### Comparison to Prior Studies

Previous studies have examined the epidemiology of non-infectious GI manifestations in PAD, primarily CVID. This includes Pikkarainen et al. who recently performed a cross-sectional analysis of the prevalence of GI disease in a Finnish population of 132 patients with CVID. The researchers found ~ 5% of participants with CVID were affected with MC, and another 7% with indeterminate colitis. The proportion increased to 10% with MC and 14% with indeterminate colitis when limiting participants to only those with CVID and GI manifestations [28]. Malamut et al. utilized a retrospective analysis in 50 French participants with CVID and known GI complications and observed that 26% had MC (23% lymphocytic colitis, 3% collagenous colitis) and another 25% had unspecified acute colitis [40]. Several other studies have estimated MC prevalence in CVID cohorts to be between 0.1 and 10% [41–44]. In contrast, no prior study has investigated the prevalence of CVID in a cohort of those already diagnosed with MC. We examined the prevalence of PAD in MC and found this to be 0.4%, and the prevalence of CVID to be specifically 0.1%. Within the histologic subtypes of MC, the prevalence of CC was 0.1%, and LC was 0.3% in those with PAD. Interestingly, the prevalence of PAD in males with MC was 0.53%, as compared to 0.30% in females. Non-infectious, inflammatory GI disease in other PAD has been less well studied, other than the established link between IgA deficiency and celiac disease [45]. Therefore, our nationwide study that examines the association between PAD and its subtypes and the risk of MC is the first to present population estimates of this relationship.

### Biological Mechanism

Our findings are biologically plausible. Patients with antibody deficiency are at increased risk for autoinflammatory complications. Non-infectious, inflammatory manifestations in CVID are associated with T- and B-cell dysfunction [17, 46]. GI inflammation in CVID may have unique inflammatory drivers as compared to IBD broadly, with increased interferon (IFN)-gamma and IL-12 [26]. Importantly, immunophenotyping of those with inflammatory GI and liver complications in CVID demonstrate peripheral and tissue aggregates of CD3 + CD8 + T cells, a hallmark histologic finding in MC [28, 40, 47]. There is also a growing body of evidence that the epithelial barrier function and gut microbiota may play a role in inflammatory manifestations in patients with CVID. Recently, Ho et al. eloquently demonstrated impaired epithelial integrity in CVID patients with non-infectious inflammatory complications through elevated markers of intestinal permeability, increased bacterial gut translocation via measurement of 16S rDNA with evidence of systemic immune activation driven by an increased interferon (IFN)-gamma signature [48]. These changes may therefore predispose individuals with PAD to MC. Unfortunately, gut microbiota changes in PAD have yet to be fully examined [49]. But several studies in specific subtypes of PAD, such as selective IgA deficiency, have shown decreased diversity and increased presence of pathogenic bacteria, which may in turn increase risk of MC [50].

We also note that a stronger association between PAD and specifically CVID, and risk of MC among males may at least in part be related to the earlier age of disease onset and more severe immunophenotype, specifically a lower mean IgM level and lower proportion of isotype-switched memory B cells in this population [51]. This association has not been previously reported with other systemic and organ-specific autoimmune complications [19, 24]. Further studies are however needed to replicate our findings and to elucidate potential biological mechanisms underlying these associations.

### Strength and Limitations

Our study has several strengths that are worth noting. First, given the population-based representation and prospectively collected data, our findings are unlikely to be prone to selection and recall biases. This is particularly relevant with regard to studies of immunodeficiency disorders that often rely on recruitment from tertiary or referral academic centers. Population estimates of CVID using ICD-10 coding from Swedish registry data, estimate prevalence in 2021 to be 1:27,000 [52]. These values corroborate with our sample of population controls with immune deficiency used in this study. Secondly, the outcome measure in this study, MC, along with variables used in stratified and sub-analyses have been extensively validated, with a high positive predictive value [30, 31, 36]. Inflammatory GI disease in individuals with PAD, specifically CVID, is known to be heterogenous, and histopathologic particularities may not be captured in registry data. As mentioned previously, within CVID, a large proportion of colitis appears to be nonspecific [28, 40]. To further limit misclassification we completed additional validation of MC cases in those with PAD via direct review of pathology reports and showed that even in this group, diagnosis of MC was highly accurate. Linkage to the multiple Swedish registries allowed us to conduct a number of exploratory and sensitivity analyses to check for the robustness of these associations.

Several study limitations should be noted. We did not have access to individual data detailing immunophenotyping, and thus, were unable to confirm each PAD case using established criteria [17]. However we note that participants were diagnosed in subspecialty clinics, and diagnostic coding has been demonstrated to have excellent positive predictive values (85%-95%) in the Swedish Patient Register [36]. The impact of misclassification of PAD was further minimized given a number of sensitivity analyses that should have increased the diagnostic accuracy. When conducting stratified analyses, given the limited sample size, we did not have enough power to examine risk in subtypes of PAD (CVID and other primary antibody deficiency), or whether specific types of infection modified this relationship. We noted a robust association between IgA deficiency and MC, although this may have been related to the low prevalence of IgA deficiency in control participants (0.009%), as compared to established Swedish general population estimates. This may be explained by the particular demographic characteristics (i.e., predominantly older women) of the study control group, or how IgA deficiency was measured (screening compared to assigned ICD-10 diagnosis) [53, 54]. Because of small sample size, we were also unable to adjust for use of medications (including prolonged antibiotics, immunosuppression, proton pump inhibitors (PPIs), and non-steroidal anti-inflammatory drugs (NSAIDs). B-cell depleting agents, such as ritxumab, are associated with both antibody deficiency and microscopic colitis [55]. PPIs, NSAIDs, and immune checkpoint inhibitors are also associated with MC, and theoretically could affect risk of autoimmune complications in PAD through alterations in intestinal permeability and the microbiome [12, 56]. Finally, the relationship between PAD and MC was attenuated, although remained significant, when restricting the population to those who started immunoglobulin replacement prior to MC diagnosis. This may be related to lower rates of GI or sinupulmonary infections or correction of immunodeficiency. Nevertheless, further details regarding the use of immunoglobulin replacement were limited, hence, the significance of this finding remains unclear.

Here, we show that PAD and particularly CVID is associated with a significantly increased risk of MC, particularly in males. Current, clinical guidelines on the clinical approach to MC involve withdrawal of potential culprit medications, and assessment of other causes of diarrhea but do not discuss predominant antibody deficiency [57, 58]. Furthermore, while the standard of care for treatment of MC involves the use of budesonide, there are no current FDA-approved therapies [59]. We propose a new endotype of MC that is male-predominant and associated with primary immunodeficiency. Clinicians should be aware of this relationship and consider a workup for antibody deficiency in males with MC and a history of recurrent infections.

## Supplementary Information

Below is the link to the electronic supplementary material.

Supplementary file1 (DOCX 28.5 KB)

## Data Availability

The data from this study are not available due to legal and ethical reasons stipulated by Swedish regulations.

## Abbreviations

CI: Confidence interval
CC: Collagenous colitis
CVID: Common variable immune deficiency
GI: Gastrointestinal
IBD: Inflammatory bowel disease
LC: Lymphocytic colitis
MC: Microscopic colitis
NSAID: Nonsteroidal anti-inflammatory drug
PAD: Predominant antibody deficiency
PPI: Proton pump inhibitor
OR: Odds ratio
