# Supplementary material for: Predominant Antibody Deficiency and Risk of Microscopic Colitis: a Nationwide Case–Control Study in Sweden
Source: J Clin Immunol. 2023 May 10;43(6):1426–35. doi: 10.1007/s10875-023-01499-3 (PMC10353958; doi:10.1007/s10875-023-01499-3)
Supplement: Supplementary file 1 — Supplementary file1 (DOCX 28.5 KB) [file 10875_2023_1499_MOESM1_ESM.docx]

**Supplemental Tables:**

| **Table S1. Definition of Predominant Antibody Deficiency Syndromes** | | |
| --- | --- | --- |
| Predominant Antibody Deficiencies  (IUIS Categories) | Sub-category | ICD-10 code |
| CVID + Hereditary Hypogammaglobulinemia (‘**CVID’**) | Hereditary hypogammaglobulinemia:  CVID with predominant abnormalities of B-cell numbers and function  CVID with predominant immunoregulatory T-cell disorders  CVID with autoantibodies to B- or T- cells  Other CVID  CVID, unspecified | D80.0  D83.0  D83.1  D83.2  D83.8  D83.9 |
| Other Predominant Antibody Deficiencies (‘**Other PAD’**) | Nonfamilial hypogammaglobulinemia  Selective deficiency of IgA  Selective deficiency of IgG  Selective deficiency of IgM  Immunodeficiency with increased IgM  Antibody deficiency with near-normal immunoglobulins or with hyperimmunoglobulinemia  Transient hypogammaglobulinemia of infancy  Other immunodeficiencies with predominantly antibody defects  Immunodeficiency with predominantly antibody defects, unspecified | D80.1  D80.2  D80.3  D80.4  D80.5  D80.6  D80.7  D80.8  D80.9 |

*CVID: common variable immunodeficiency, PAD: predominant antibody deficiency, ICD: International Classification of Diseases

| **Table S2. ATC codes for immunoglobulin administration** | | |
| --- | --- | --- |
| **Category** | **Details** | **ATC code** |
| **Immunoglobulin Replacement** | Immunoglobulins, normal human, for extravascular administration  Immunoglobulins, normal human, for intravascular administration | J06BA01  J06BA01 |

*ATC: Anatomical Therapeutic Chemical

| **Table S3. ICD codes in gastrointestinal and sinopulmonary infections** | | | | |
| --- | --- | --- | --- | --- |
| **Infection** | **ICD-7 code** | **ICD-8 code** | **ICD-9** | **ICD-10 code** |
| **Gastrointestinal (Any)** | 471; 513 | 461, 503 | 461, 473 | A00-A09 |
| **Sinusitis** | 040-049 | 000-009 | 001-009 | J01, J32 |
| **Pneumonia** | 490-93 | 481-86 | 481-86 | J13-J19 |
| **Otitis** | 391, 392 | 380-382 | 381, 382 | H65, H66 |

*ICD: International Classification of Diseases

| **Table S4. ICD codes for different immune-mediated diagnoses** | | | | |
| --- | --- | --- | --- | --- |
| **Disease** | **ICD-7 code** | **ICD-8 code** | **ICD-9 code** | **ICD-10 code** |
| *Diabetes* | 260 | 250 | 250 | E10-14 |
| *Psoriasis* | 706,09 | 696 (969,30 excluded) | 696A-C + E-W | L40 |
| *SLE* | 456,20 | 734,1 | 710A | M32.1; M32.8; M32.9 |
| *Rheumatoid arthritis* | 722,00 + 722,01 | 712,3; 714,93 | 714 | M05; M06; M08; M09; M12.3 |
| *Thyroiditis* | 254 | 245 | 245A-X | E06 |
| *Hyperthyroidism* | 252.00, 252.01, 252.02, 254.00; | 242 | 242 | E05 |
| *Sarcoidosis* | 138,00 to 138,04 | 135 | 135 | D86 |
| *Primary biliary cirrhosis* |  |  | 571G | K74.3 |
| *ANCA vasculitis and other vasculitis* |  | 446 | 446 | M31 |
| *Ankylosing spondylitis* |  |  |  | M45.9 |
| *Autoimmune hepatitis* |  | 573,0x (chronic hepatitis)  571,9x | 573D + 573E | K75.4; K75.5; K75.9 |
| *Primary sclerosing cholangitis* |  |  |  | K83.0A |

*ICD: International Classification of Diseases, SLE: systemic lupus erythematosus, ANCA: anti-neutrophil cytoplasmic antibody

| **Table S5. Causes of secondary hypogammaglobulinemia** | | |
| --- | --- | --- |
| **Category** | **ICD-9 code** | **ICD-10 code** |
| **Cancer (lymphoid, hematopoietic)**  **Nephrotic syndrome**  **Intestinal malabsorption**  **Burns (excluding those < 10% of body surface area)**  **Malnutrition**  **Human immunodeficiency virus** | 200-209  581  579.9  948.1-948.99  260-263.9  042 | C81-C96  N04  K90.9  T31.1-T31.99  E40-E46  B20 |

*ICD: International Classification of Diseases

| **Table S6: Baseline characteristics of cases of microscopic colitis and their unaffected siblings** | | |
| --- | --- | --- |
|  | | |
|  | Microscopic Colitis | Sibling |
|  | | |
|  | n [%] | n [%] |
| Total | 7123 [100.0] | 13759 [100.0] |
| Sex, female | 5162 [72.5] | 6897 [50.1] |
| Age at start of follow-up, years |  |  |
| Mean [SD] | 54.4 [15.1] | 54.4 [14.5] |
| Median [IQR] | 58.0 [45.0-66.0] | 57.0 [46.0-65.0] |
| < 50 years | 2285 [32.1] | 4308 [31.3] |
| ≥ 50 years | 4838 [67.9] | 9451 [68.7] |
| Time from PAD to MC/index date, years |  |  |
| Mean [SD] | 7.6 [6.6] | 5.6 [5.0] |
| Median [IQR] | 6.0 [2.4-10.0] | 3.4 [2.4-7.9] |
| < 1 years | 4 [13.3] | 2 [10.5] |
| 1 < 5 years | 9 [30.0] | 9 [47.4] |
| ≥ 5 years | 17 [56.7] | 8 [42.1] |
| Year of start of follow-up |  |  |
| 1997 - 2001 | 544 [7.6] | 1094 [8.0] |
| 2002 - 2006 | 1363 [19.1] | 2708 [19.7] |
| 2007 - 2011 | 2440 [34.3] | 4773 [34.7] |
| 2012 - 2017 | 2776 [39.0] | 5184 [37.7] |
|  | | |

*IQR: interquartile range, MC: microscopic colitis, PAD: predominant antibody deficiency, SD: standard deviation

| **Table S7: Association between predominant antibody deficiency and risk of microscopic colitis subtypes** | | | |
| --- | --- | --- | --- |
|  | | | |
|  | **CC** Exposed (N) | Controls Exposed (N) | aOR [95% CI] |
|  | | | |
|  |  |  |  |
| PAD | 14/4410 | 9/20938 | 7.16 [3.10-17.35] |
| CVID | 2/4410 | 0/20938 | - |
| Other PAD | 12/4410 | 9/20938 | 6.02 [2.52-14.91] |
|  | **LC** Exposed (N) | Controls Exposed (N) | aOR [95% CI] |
| PAD | 36/9241 | 21/44559 | 7.46 [4.37-13.03] |
| CVID | 8/9240 | 2/44555 | 16.35 [4.05-108.95] |
| Other PAD | 27/9240 | 19/44555 | 6.29 [3.50-11.53] |

| **Table S8: Association between common variable immunodeficiency and microscopic colitis across selected strata** | | | |
| --- | --- | --- | --- |
|  | | | |
|  | MC Exposed (N) | Controls Exposed (N) | aOR [95% CI] |
|  | | | |
| Total | 10 | 2 | 21.00 [5.47-137.42] |
| Sex |  |  |  |
| Males | 4/3779 | 0/18034 | NA |
| Females | 6/9871 | 2/47463 | 11.75 [2.66-81.00] |
| Age at MC diagnosis/matching, years |  |  |  |
| ≤ 50 years | 6/3383 | 0/16756 | NA |
| > 50 years | 4/10267 | 2/48741 | 8.07 [1.55-58.81] |
| Years exposed before MC diagnosis/matching |  |  |  |
| < 1 years | 0/13609 | 0/65469 | NA |
| 1 < 5 years | 1/13617 | 0/65479 | 15149.73 [0.08-NA] |
| ≥ 5 years | 9/13626 | 2/65483 | 19.45 [4.96-128.29] |
| Year of MC diagnosis/matching |  |  |  |
| 1997 - 2001 | 0/1329 | 0/6458 | NA |
| 2002 - 2006 | 2/3111 | 1/14994 | 6.97 [0.65-152.28] |
| 2007 - 2011 | 2/4592 | 0/22007 | NA |
| 2012 - 2017 | 6/4618 | 1/22038 | 27.37 [4.62-518.98] |

*CI: confidence interval, MC: microscopic colitis, NA: not applicable, OR: odds ratio

**†**aOR adjusted for age, sex, year, county, and immune-mediated disease

| **Table S9: Association between other predominant antibody deficiency and microscopic colitis across selected strata** | | | |
| --- | --- | --- | --- |
|  | | | |
|  | MC Exposed (N) | Controls Exposed (N) | aOR [95% CI] |
|  | | | |
|  |  |  |  |
| Total | 39 | 28 | 6.16 [3.79-10.14] |
| Sex |  |  |  |
| Males | 16/3779 | 3/18034 | 25.35 [8.40-109.34] |
| Females | 23/9871 | 25/47463 | 3.95 [2.21-7.01] |
| Age at MC diagnosis/matching, years |  |  |  |
| ≤ 50 years | 11/3383 | 6/16756 | 8.36 [3.13-24.56] |
| > 50 years | 28/10267 | 22/48741 | 5.56 [3.17-9.86] |
| Years exposed before MC diagnosis/matching |  |  |  |
| < 1 years | 8/13609 | 2/65469 | 19.82 [4.95-131.61] |
| 1 < 5 years | 15/13617 | 12/65479 | 5.72 [2.66-12.53] |
| ≥ 5 years | 16/13626 | 14/65483 | 4.68 [2.26-9.79] |
| Year of MC diagnosis/matching |  |  |  |
| 1997 - 2001 | 0/1329 | 1/6458 | NA |
| 2002 - 2006 | 9/3111 | 9/14994 | 4.39 [1.70-11.36] |
| 2007 - 2011 | 9/4592 | 8/22007 | 5.32 [2.02-14.26] |
| 2012 - 2017 | 21/4618 | 10/22038 | 9.05 [4.33-20.20] |

*CI: confidence interval, MC: microscopic colitis, NA: not applicable, OR: odds ratio,

**†**aOR adjusted for age, sex, year, county, and immune-mediated disease
